# FTO knockdown in rat ventromedial hypothalamus does not affect energy balance

**DOI:** 10.14814/phy2.12152

**Published:** 2014-12-11

**Authors:** Margriet A. van Gestel, Loek E. Sanders, Johannes W. de Jong, Mieneke C. M. Luijendijk, Roger A. H. Adan

**Affiliations:** 1 Brain Center Rudolf Magnus, Department of Translational Neuroscience, University Medical Center Utrecht, Utrecht, The Netherlands

**Keywords:** Feeding, FTO, obesity, ventromedial hypothalamus

## Abstract

Single nucleotide polymorphisms (SNPs) clustered in the first intron of the fat mass and obesity‐associated (*FTO*) gene has been associated with obesity. FTO expression is ubiquitous, with particularly high levels in the hypothalamic area of the brain. To investigate the region‐specific role of *FTO*, AAV technology was applied to knockdown *FTO* in the ventromedial hypothalamus (VMH). No effect of *FTO* knockdown was observed on bodyweight or parameters of energy balance. Animals were exposed twice to an overnight fast, followed by a high‐fat high‐sucrose (HFHS) diet for 1 week. *FTO* knockdown did not result in a different response to the diets. A region‐specific role for *FTO* in the VMH in the regulation of energy balance could not be found.

## Abstract (precis)

Knocking down expression of the obesity‐associated gene FTO in rat ventromedial hypothalamus did not affect energy balance.

## Introduction

Overweight and obesity are increasingly important health problems worldwide. The World Health Organization reports that 1.4 billion adults are overweight and approximately one‐third of them are obese. During 1980 and 2008, obesity rates nearly doubled (Finucane et al. 2011). Obesity has been implicated as a major risk factor for cardiovascular diseases (Garrison et al. 1987; Manson et al. 1995; Ogden et al. 2007) and diabetes (Field et al. 2001; Oguma et al. 2005). Furthermore, obesity was associated with depression (Luppino et al. 2010). An environment that promotes high caloric food intake and discourages physical activity contributes to the occurrence of obesity. Obesity‐associated genes might explain why individuals respond differently to this obesogenic environment. Indeed, family, twin and adoptions studies point to a strong genetic basis for the development of obesity (Stunkard et al. 1986a,b; MacDonald 1990; Maes et al. 1997).

In 2007, studies confirmed the fat mass and obesity‐associated (*FTO*) gene as the first genome‐wide association study (GWAS)‐identified obesity susceptibility gene (Dina et al. 2007; Frayling et al. 2007; Scuteri et al. 2007). Common variants in the first intron of the *FTO* gene were associated with an increase in body mass index (BMI) of approximately 0.4 kg/m^2^ per risk allele (Frayling et al. 2007). Variations in the *FTO* gene seem to influence energy balance by increased energy intake (Cecil et al. 2008; Speakman et al. 2008; Timpson et al. 2008; Haupt et al. 2009; Tanofsky‐Kraff et al. 2009; Wardle et al. 2009) and not by decreased physical activity (Berentzen et al. 2008; Do et al. 2008; Speakman et al. 2008; Goossens et al. 2009; Hakanen et al. 2009; Haupt et al. 2009; Wardle et al. 2009; Liu et al. 2010). FTO was identified as a 2‐oxoglutarate‐dependent nucleic acid demethylase and is involved in the demethylation of single‐stranded DNA and RNA (Gerken et al. 2007; Jia et al. 2008). It is suggested that FTO may regulate transcription of genes involved in energy balance by demethylation (Gerken et al. 2007).

FTO is widely expressed throughout the brain, especially in the hypothalamic arcuate (ARC), paraventricular, dorsomedial (DMH), and ventromedial (VMH) nuclei (Gerken et al. 2007; McTaggart et al. 2011). In this study, we focused on the role of *FTO* on energy balance in the VMH, a hypothalamic nucleus involved in obesity, fear, and female reproductive behavior (Brobeck et al. 1943; Mathews and Edwards 1977; Satoh et al. 1997; Trogrlic et al. 2011). A microRNA‐expressing AAV was injected into the VMH of rats and bodyweight, food intake, locomotor activity, and body temperature were monitored. No effect of *FTO* knockdown was found on bodyweight or parameters of energy balance. We previously showed that exposure to a restricted feeding schedule results in increased expression of FTO in the ARC and the VMH (Boender et al. 2012). To examine the effect of fasting on bodyweight and food intake, animals with *FTO* knockdown were exposed to an overnight fast twice. We did not observe an effect of fasting on bodyweight or on refeeding after restriction. Finally, a high‐fat high‐sucrose (HFHS) diet was introduced to the animals. Again, no differences were seen between the controls and the VMH *FTO* knockdown animals in their response to the HFHS diet. *FTO* in the VMH seems to have no impact on bodyweight or energy balance.

## Material and Methods

### Cell lines

Human embryonic kidney (HEK) 293T cells were maintained at 37°C with 5% CO_2_ in growth medium (Dulbecco's modified Eagle medium, DMEM) (Invitrogen, Carlsbad, CA) supplemented with 10% fetal calf serum (FCS) (Lonza, Basel, Switzerland), 2 mmol/L glutamine (PAA, Cölbe, Germany), 100 units/mL penicillin (PAA), 100 units/mL streptomycin (PAA), and nonessential amino acids (PAA).

### Construction of plasmids

A FTO‐Renilla fusion plasmid was constructed as previously described (Van Gestel et al. 2014). Experiments were conducted using miRNAs targeting *FTO,* a control miRNA targeting *Hcrtr1* and a control miRNA targeting Firefly Luciferase. pAAVs‐expressing miRNAs were generated using the Gateway cloning technology (Invitrogen) as previously described (White and Nolan 2011). Briefly, miRNA sequences targeting *FTO* and *Hcrtr1* were designed using the ‘Block‐iT RNAi Designer’ (Invitrogen) (Table 1). The oligos were annealed and ligated into the synthetic intron of PSM155 (Du et al. 2006). A cassette containing the intronic miRNA upstream of enhanced green fluorescent protein (EGFP) was then amplified using B3 and B4 primers and recombined to generate the entry vectors pENTR‐R4‐miFTO1‐EGFP‐R3, pENTR‐R4‐miFTO2‐EGFP‐R3, pENTR‐R4‐miFTO3‐EGFP‐R3, and pENTR‐R4‐miHcrtr1‐EGFP‐R3. Each entry vectors was recombined with pENTR‐L1‐ESYN‐L4, pENTR‐L3‐oPRE‐L2, and pAAV‐R1‐R2 to generate pAAV‐ESYN‐miFTO1‐EGFP (pAAV‐miFTO#1), pAAV‐ESYN‐miFTO2‐EGFP (pAAV‐miFTO#2), pAAV‐ESYN‐miFTO3‐EGFP (pAAV‐miFTO#3), and pAAV‐ESYN‐miHcrtr1‐EGFP (pAAV‐miHcrtr1). pAAV‐miLuc was a kind gift of M.F. Nolan (White and Nolan 2011).

**Table 1.** Overview of oligonucleotides used in this study. Overview of oligonucleotides that were used to obtain miRNAs targeting *FTO* and *Hcrtr1* mRNA and to perform a qPCR.

miFTO#1	Forward	TGCTGTTTAGGATATTTCAGCTGCCAGTTTTGGCCACTGACTGACTGGCAGCTAATATCCTAAA
	Reverse	CCTGTTTAGGATATTAGCTGCCAGTCAGTCAGTGGCCAAAACTGGCAGCTGAAATATCCTAAAC
miFTO#2	Forward	TGCTGTTAAGGTCCACTTCATCATCGGTTTTGGCCACTGACTGACCGATGATGGTGGACCTTAA
	Reverse	CCTGTTAAGGTCCACCATCATCGGTCAGTCAGTGGCCAAAACCGATGATGAAGTGGACCTTAAC
miFTO#3	Forward	TGCTGAGCAAAGTCACGTTGTAGGCTGTTTTGGCCACTGACTGACAGCCTACAGTGACTTTGCT
	Reverse	CCTGAGCAAAGTCACTGTAGGCTGTCAGTCAGTGGCCAAAACAGCCTACAACGTGACTTTGCTC
miHcrtr	Forward	TGCTGATGAGAACCCACTCGTACTGCGTTTTGGCCACTGACTGACGCAGTACGTGGGTTCTCAT
	Reverse	CCTGATGAGAACCCACGTACTGCGTCAGTCAGTGGCCAAAACGCAGTACGAGTGGGTTCTCATC
GFP	Forward	CACAGACTTGTGGGAGAAGC
	Reverse	CCCCTGAACCTGAAACATAAA
FTO#1 qPCR	Forward	GAGCGGGAAGCTAAGAAACTG
	Reverse	CTTGTGCAGTGTGAGAAAGGC
FTO#3 qPCR	Forward	CGCATGTCAGACCTTCCTCA
	Reverse	AGTCACGTTGTAGGCTGCTC
CycA qPCR	Forward	AGCCTGGGGAGAAAGGATT
	Reverse	AGCCACTCGTCTTGGCAGT

### Luciferase assay

HEK293T cells in a 24‐well plate were transfected with 5 ng pcDNA4/TO‐luc, 500 ng pBabe‐FTO‐Renilla, and 1500 ng pAAV‐miFTO or pAAV‐miHcrtr1 using polyethylenimine (PEI) (Polysciences, Eppelheim, Germany). Three days after transfection, cells were lysed in passive lysis buffer and analyzed with a dual luciferase reporter assay according to manufacturer's protocol (Promega, Madison, WI). Firefly and Renilla luciferase activity were assessed; values were corrected for transfection efficiency using Firefly Luciferase activity and normalized to pAAV‐miHcrtr1 knockdown.

### Virus production and purification

Virus was generated and purified as previously described (De Backer et al. 2010). Briefly, HEK293T cells were co‐transfected with pAAV‐miRNA and pDP1 (Plasmid Factory, Bielefeld, Germany) in fifteen 15 × 15 cm dishes using PEI. Sixty hours after transfection, cells were collected, pelleted, and resuspended in ice‐cold buffer (150 mmol/L NaCl, 50 mmol/L Tris, pH 8.4). Cells were lysed by three freeze–thaw cycles and incubated for 30 min at 37°C with 50 U/mL benzonase (Sigma, Zwijndrecht, the Netherlands). The lysate was loaded onto a 15%, 25%, 40%, and 60% iodixanol gradient. After centrifugation at 500,000 × g for 60 min at 18°C, the 40% layer was extracted and used for ion‐exchange chromatography. AAV positive fractions were determined by quantitative PCR (qPCR) on GFP (Table 1) and concentrated using an Amicon Ultra 15 mL filter (Millipore, Amsterdam, the Netherlands). Titer was determined by qPCR on GFP.

### Animal studies

Male Wistar rats (Charles River, Germany) of 220–250 g were housed in filter top cages in a temperature‐ and humidity‐controlled room (temperature 21 ± 2°C and humidity 55 ± 5%) with a 12 h light/dark cycle. Animals had ad libitum access to chow and water. After 1 week of acclimatization, pAAV‐miRNA was administered stereotactically in the VMH. Animals were exposed to an overnight fast for 16 h twice in the sixth week after surgery by food restricting them from 1700 h to 900 h. All animals had to be at their original weight before exposure to the second overnight fast. Refeeding was measured by calculating cumulative chow intake after 2, 4, 8, and 24 h. In the seventh week after surgery, animals were exposed to a HFHS diet. The HFHS diet consisted of ad libitum access to chow, saturated fat (Vandemoortele, Eeklo, Belgium) and a 30% sucrose solution (Suiker Unie, Oud Gastel, the Netherlands). All experimental procedures were approved by the Committee for Animal Experimentation of the University of Utrecht (Utrecht, the Netherlands).

### Surgical procedures

Rats were anesthetized using fentanyl/fluanisone and midazolam and mounted onto a stereotaxic apparatus. Virus was administered by placing a syringe needle into the VMH (coordinates from Bregma: −2.1 AP, +1.5 ML, −9.9 DV, at a 5° angle). A total of 1 *μ*L virus (1 × 10^12^ genomic copies/mL) were injected at a rate of 0.2 *μ*L/min. Rats received a transmitter in the abdominal cavity for the recording of locomotor activity and body temperature (TA10TA‐F40, Data Science International, New Brighton, MN).

### In situ hybridization (ISH)

For the ISH, cryostat sections of 20 *μ*m thickness from fresh, frozen brains were mounted onto slides. Sections were fixed in 4% paraformaldehyde for 20 min, washed in phosphate‐buffered saline, acetylated for 10 min and washed again. The following steps differed between the ISH and the locked nucleic acid (LNA) ISH.

For the ISH, sections were prehybridized in hybridization solution (50% formamide, 5 × SSC, 5 × Denhardts, 250 *μ*g/mL tRNA Baker's yeast, 500 *μ*g/mL sonicated salmon sperm DNA) for 2 h at room temperature. The hybridization solution containing 400 ng/mL 720‐bp long digoxigenin (DIG)‐labeled EGFP riboprobe (antisense to NCBI gene DQ768212) was then applied to the slides followed by overnight incubation at 68°C. After a quick wash in 68°C prewarmed 2 × SSC, slides were transferred to 68°C prewarmed 0.2 × SSC for 2 h. After blocking for 1 h with 10% FCS in B1 (0.1 mol/L Tris pH 7.5/0.15 mol/L NaCl), DIG was detected with an alkaline phosphatase‐labeled antibody (1:5000, Roche, Mannheim, Germany) after overnight incubation at RT using NBT/BCIP as a substrate. Sections were dehydrated in ethanol, cleared in xylene, and embedded in Entellan.

The LNA ISH hybridization is performed as previously described (Kan et al. 2012). Briefly, sections were prehybridized in hybridization solution (50% formamide, 5 × SSC, 5 × Denhardts, 200 mg/mL tRNA Baker's yeast, 500 mg/mL sonicated salmon sperm DNA, 0.02 g/mL Roche blocking reagent) for 1 h at RT. Hybridization was performed with 10 nmol/L double‐DIG (3′ and 5′)‐labeled LNA probe for human miR‐124 (Exiqon, Vedbaek, Denmark) for 2 h at 55°C. After a quick wash in 60°C prewarmed 5 × SSC, slides were transferred to 60°C prewarmed 0.2 × SSC for 2 h. After blocking for 1 h with 10% FCS in B1 (0.1 mol/L Tris pH 7.5/0.15 mol/L NaCl), DIG was detected with an alkaline phosphatase‐labeled antibody (1:2500, Roche) after overnight incubation at RT using NBT/BCIP as a substrate. Slides were further processed for immunohistochemistry.

### RNA isolation and qPCR in vivo knockdown

The VMH was dissected from fresh frozen cryostat sections. RNA was extracted by adding 0.5 mL Trizol to the tissue. After 5 min incubation at RT, 100 *μ*L chloroform was added followed by 2 min incubation at RT. After centrifugation for 15 min at 16,200 × g, RNA was precipitated from the aqueous layer by adding 0.25 mL isopropanol. After 10 min incubation at RT, samples were centrifuged for 10 min at 13,000 rpm. Next, the pellet was washed with 0.5 mL 100% ethanol. After another centrifugation step, the pellet was dissolved in water. Expression of FTO was detected using quantitative PCR using primers for each miRNA sequence and a housekeeping gene, CycA (Table 1). The difference between Ct values of FTO and CycA was calculated for each sample. Next, the difference between the AAV‐mirFTO#1/2 samples and the AAV‐mirLuc samples was calculated to determine fold change.

### Statistical analyses

All data were presented as means ± SEM. Statistical analyses were performed using GraphPad Prism 5 software (GraphPad Software, Inc., La Jolla, CA). A *P*‐value of <0.05 was considered to be significant.

## Results

### In vitro knockdown efficiency

**Figure 1.**
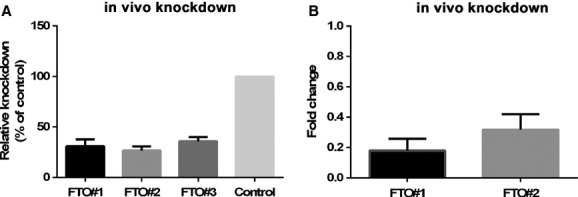
*In vitro* and in vivo knockdown efficiency of FTO constructs. In vitro knockdown efficiency of a cDNA FTO‐Renilla fusion construct by pAAV‐miFTO(1‐3) relative to the control pAAV‐miHcrtr1. pAAV‐miFTO#1 and pAAV‐miFTO#2 were selected for in vivo use based on their silency efficacy of 69% and 74%, respectively (A). In vivo knockdown efficiency of pAAV‐FTO#1 and pAAV‐FTO#2 in the VMH as measured by qPCR for FTO (B).

Three different miRNA sequences targeting *FTO* were cloned into an AAV2 vector and their in vitro knockdown efficiency was determined using a dual luciferase assay. pAAV‐miHcrtr1 was used as a control. pAAV‐miFTO#1 and pAAV‐miFTO#2 were selected for further study because of their 69% and 74% silencing efficacy, respectively (Fig. 1A).

### In vivo knockdown efficiency and hypothalamic injections

**Figure 2.**
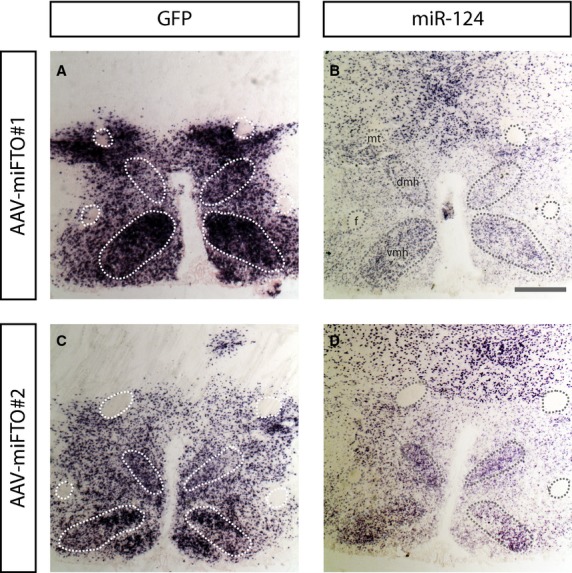
VMH transduction and miR‐124 expression. Both AAV‐miFTO#1 and #3 efficiently transduced the VMH (A and C). MiR‐124 expression was not affected by transduction (B and D). mt = mammillary tract; *f* = fornix; dmh = dorsomedial hypothalamus; vmh = ventromedial hypothalamus. Scale bar: 500 *μ*m

The plasmids were encapsidated into an AAV1 coat and AAV‐miFTO#1 (*n* = 8), AAV‐miFTO#2 (*n* = 8) and AAV‐miLuc (*n* = 8) were stereotactically injected into the ventromedial hypothalamus. After 5 weeks, their brains were analyzed for in vivo knockdown efficiency and the placement of the hypothalamic injection. In vivo knockdown of *FTO* in the VMH was confirmed by qPCR. Due to technical problems, tissue from four animals could not be studied for knockdown efficiency. AAV‐miFTO#1 (*n* = 6) and AAV‐miFTO#2 (*n* = 8) decreased *FTO* mRNA levels by 82% and 68% compared to controls (*n* = 6), respectively (Fig. 1B). The viral vector contained a GFP cassette allowing the transduced area to be precisely identified. An ISH to detect GFP mRNA expression was used to analyze transduction efficiency. Figure 2A and C represent typical examples of injections targeting the ventromedial hypothalamus. To ensure that the hypothalamic injections did not result in toxicity due to oversaturation of the microRNA pathway as observed after AAV‐mediated short hairpin RNA expression in the VMH (Van Gestel et al. 2014), we performed a miRNA 124 LNA ISH, as described previously (Van Gestel et al. 2014). No decreased miRNA 124 levels were observed after AAV transduction (Fig. 2B and D), indicating no oversaturation of the miRNA pathway.

### Bodyweight and energy balance

**Figure 3.**
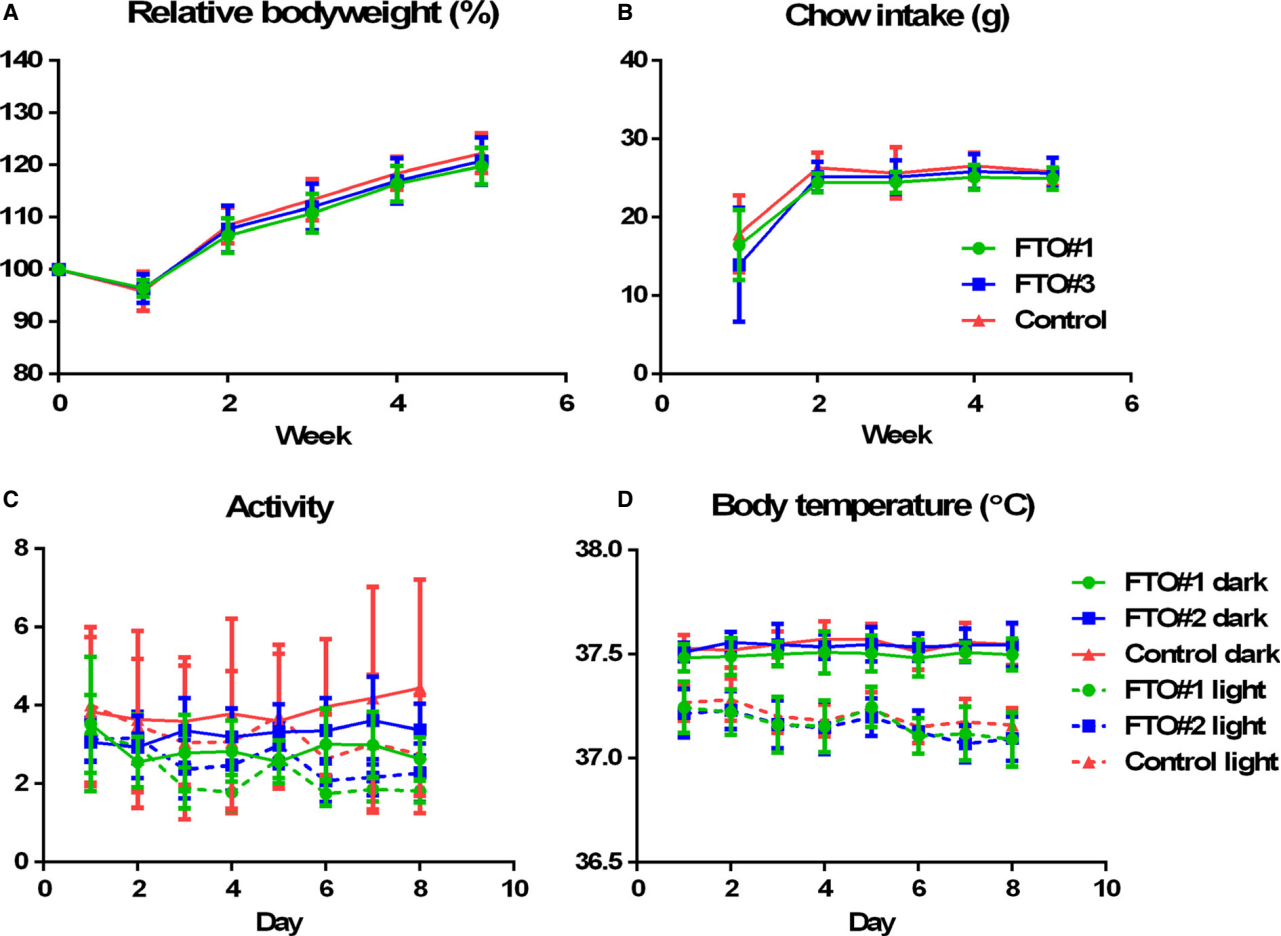
Bodyweight and parameters of energy balance after FTO knockdown in the VMH. Relative bodyweight (A) and chow intake (B) were measured for 5 weeks. No differences in relative bodyweight (two‐way ANOVA,* f* = 1.363, *P* = 0.2057) or chow intake (two‐way ANOVA,* f* = 1.215, *P* = 0.2992) were observed after *FTO* knockdown in the VMH. In the third week, locomotor activity (C) and body temperature (D) were assessed. *FTO* knockdown in the VMH did not affect locomotor activity (dark phase: two‐way ANOVA,* f* = 1.452, *P* = 0.1371; light phase: two‐way ANOVA,* f* = 0.8037, *P* = 0.6640) or body temperature (dark phase: two‐way ANOVA,* f* = 0.5881, *P* = 0.8709; light phase: two‐way ANOVA,* f* = 1.015, *P* = 0.4420) in the dark phase or in the light phase.

Groups were matched for bodyweight and chow intake prior to surgery. After injection of the AAVs into the VMH, bodyweight and chow intake were measured for 5 weeks. No effect of *FTO* knockdown was found on bodyweight (two‐way ANOVA, *f* = 1.363, *P* = 0.2057) or chow intake (two‐way ANOVA, *f* = 1.215, *P* = 0.2992) (Fig. 3A and B). Locomotor activity and body temperature were measured in the third week after surgery and used as a measure for energy expenditure. No differences in locomotor activity (dark phase: two‐way ANOVA, *f* = 1.452, *P* = 0.1371; light phase: two‐way ANOVA, *f* = 0.8037, *P* = 0.6640) or body temperature were observed (dark phase: two‐way ANOVA, *f* = 0.5881, *P* = 0.8709; light phase: two‐way ANOVA, *f* = 1.015, *P* = 0.4420) (Fig. 3C and D).

### High‐fat high‐sucrose diet and fasting

**Figure 4.**
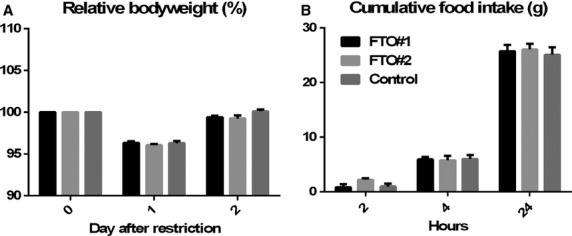
Overnight fasting. Animals were exposed to an overnight fast twice. Food was removed for 16 h from 1700 h to 900 h. Bodyweight at day 0 (the day before the overnight fast) was set at 100%. Fasting had no effect on relative bodyweight (two‐way ANOVA,* f* = 1.845, *P* = 0.1393) (A). Cumulative food intake was measured 2, 4, and 24 h after refeeding. No effect of *FTO* knockdown in the VMH was found on refeeding (two‐way ANOVA,* f* = 0.3633, *P* = 0.8329) (B).

**Figure 5.**
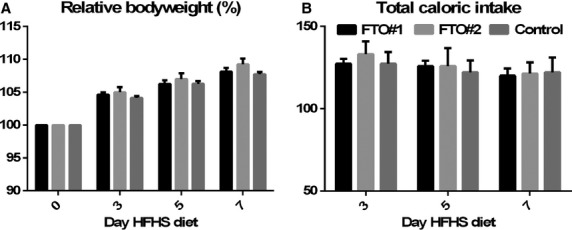
HFHS diet. In the seventh week after surgery, animals received a HFHS diet for 1 week. *FTO* knockdown in the VMH had no implications for bodyweight (two‐way ANOVA,* f* = 0.9387, *P* = 0.4743) (A) or total caloric intake (two‐way ANOVA,* f* = 0.4805, *P* = 0.7499) (B) on a HFHS diet.

Animals were fasted two times for 16 h and refeeding was measured. Fasting had no effect on bodyweight (two‐way ANOVA, *f* = 1.845, *P* = 0.1393) and no differences were observed in refeeding (two‐way ANOVA, *f* = 0.3633, *P* = 0.8329) (Fig. 4A and B). To examine the effect VMH *FTO* knockdown on high caloric food intake, animals were exposed to a high‐fat high‐sucrose diet for 1 week. No effect was observed on bodyweight (two‐way ANOVA, *f* = 0.9387, *P* = 0.4743) and total caloric intake (two‐way ANOVA, *f* = 0.4805, *P* = 0.7499) (Fig. 5A and B).

## Discussion

In 2007, single nucleotide polymorphisms in the first intron of *FTO* were associated with body mass index. *FTO* is ubiquitously expressed in the brain, with high levels in the hypothalamic nuclei, which are important contributors to energy homeostasis. Previously, we have shown that VMH *FTO* is upregulated after exposure to a restricted feeding schedule. In this study, we investigated whether *FTO* knockdown in the VMH affects energy balance.

AAV‐miFTO was bilaterally injected in the VMH and the effect on energy balance was measured for 5 weeks. *FTO* knockdown in the VMH did not affect bodyweight, food intake, body temperature, or locomotor activity. Next, animals were exposed twice to an overnight fast, which did not result in differences in bodyweight or refeeding. Finally, animals received a high‐fat high‐sucrose diet for 1 week. Animals did not show a difference in caloric intake, nor was their bodyweight differently affected by the diet.

Mouse models with an (in)complete *FTO* loss‐of‐function demonstrate reduced body weight and fat mass, without a decrease in energy intake (Church et al. 2009; Fischer et al. 2009). Consistent with these findings, mice overexpressing *FTO* show increased bodyweight and fat mass on both chow and high‐fat diet (Church et al. 2010). In contrast to the *FTO* deficient models, this effect on body weight was the result of an increase in food intake. Although it was shown that *FTO*‐mediated regulation of energy balance is located in the brain (Gao et al. 2010), *FTO* knockdown in the VMH did not result in a change in bodyweight or parameters of energy balance. Despite the very efficient in vivo knockdown of 84% in this study, it seems that *FTO* in the VMH does not affect energy homeostasis. *FTO* knockdown in the arcuate nucleus or mediobasal hypothalamus resulted in a modest reduction in food intake in the first week (Tung et al. 2010) and a reduction in food intake and bodyweight gain (McMurray et al. 2013), respectively. In this study, *FTO* knockdown was not limited to the VMH and parts of the DMH and ARC were transduced as well in some rats. In this region, *FTO* expression is highest in the VMH (Boender et al. 2012) and therefore we targeted the VMH. As we did not observe a phenotype in any of the rats, we did not find evidence for a role of *FTO* in the DMH and ARC in energy balance. However, we cannot exclude that knockdown of *FTO* in these structures might counteract the consequences of *FTO* knockdown in the VMH. The latter seems less likely considering the outcome after *FTO* knockdown in the ARC (Tung et al. 2010). Furthermore, although the virus spread could be observed throughout the whole mediobasal hypothalamus, some regions in the rostro‐caudal extent of the VMH might be spared or have an incomplete transduction. Although these regions are relatively small, theoretically these might compensate for the loss of FTO. Possibly, other factors implicated in transcription and translation can compensate for a reduction in FTO, resulting in the absence of an effect of *FTO* knockdown. Although AAV‐mediated knockdown was very effective, we cannot rule out that there are still functional levels of FTO protein.

Animals did not respond differently to exposure to overnight fasting, although we previously observed upregulation of FTO after exposure to a restricted feeding schedule. An overnight fast might not be enough for the development of a phenotype, secondary to the altered mRNA levels. Although effects of diet manipulation on *FTO* levels vary a lot in general, we cannot rule out the possibility that *FTO* knockdown in the VMH will result in a different outcome after exposure to a restricted feeding schedule.

One of the limitations of a GWA study is that SNPs are associated that may link to a more distant gene than to the nearest gene. Recent studies have shown that the obesity‐associated SNPs embedded in the first intron of *FTO* are influencing expression of different, distant genes, called *RPGRIP1L* and *IRX3*, instead of affecting *FTO* itself (Smemo et al. 2014; Stratigopoulos et al. 2014). This might explain the absence of a phenotype after *FTO* knockdown. More research needs to be conducted to clarify the role of *RPGRIP1L* and *IRX3* in obesity and their relation to FTO.

## Conflict of Interest

None declared.
